# *Vibrio cholerae* Serogroup O139: Isolation from Cholera Patients and Asymptomatic Household Family Members in Bangladesh between 2013 and 2014

**DOI:** 10.1371/journal.pntd.0004183

**Published:** 2015-11-12

**Authors:** Fahima Chowdhury, Alison E. Mather, Yasmin Ara Begum, Muhammad Asaduzzaman, Nabilah Baby, Salma Sharmin, Rajib Biswas, Muhammad Ikhtear Uddin, Regina C. LaRocque, Jason B. Harris, Stephen B. Calderwood, Edward T. Ryan, John D. Clemens, Nicholas R. Thomson, Firdausi Qadri

**Affiliations:** 1 International Centre for Diarrhoeal Disease Research Bangladesh (icddr,b), Dhaka, Bangladesh; 2 Wellcome Trust Sanger Institute, Wellcome Trust Genome Campus, Hinxton, Cambridge, United Kingdom; 3 Massachusetts General Hospital, Boston, Massachusetts, United States of America; 4 Harvard Medical School, Boston, Massachusetts, United States of America; 5 UCLA Fielding School of Public Health, Los Angeles, California, United States of America; 6 Department of Pathogen Molecular Biology, the London School of Hygiene and Tropical Medicine, London, United Kingdom; Beijing Institute of Microbiology and Epidemiology, CHINA

## Abstract

**Background:**

Cholera is endemic in Bangladesh, with outbreaks reported annually. Currently, the majority of epidemic cholera reported globally is El Tor biotype *Vibrio cholerae* isolates of the serogroup O1. However, in Bangladesh, outbreaks attributed to *V*. *cholerae* serogroup O139 isolates, which fall within the same phylogenetic lineage as the O1 serogroup isolates, were seen between 1992 and 1993 and in 2002 to 2005. Since then, *V*. *cholerae* serogroup O139 has only been sporadically isolated in Bangladesh and is now rarely isolated elsewhere.

**Methods:**

Here, we present case histories of four cholera patients infected with *V*. *cholerae* serogroup O139 in 2013 and 2014 in Bangladesh. We comprehensively typed these isolates using conventional approaches, as well as by whole genome sequencing. Phenotypic typing and PCR confirmed all four isolates belonging to the O139 serogroup.

**Findings:**

Whole genome sequencing revealed that three of the isolates were phylogenetically closely related to previously sequenced El Tor biotype, pandemic 7, toxigenic *V*. *cholerae* O139 isolates originating from Bangladesh and elsewhere. The fourth isolate was a non-toxigenic *V*. *cholerae* that, by conventional approaches, typed as O139 serogroup but was genetically divergent from previously sequenced pandemic 7 *V*. *cholerae* lineages belonging to the O139 or O1 serogroups.

**Conclusion:**

These results suggest that previously observed lineages of *V*. *cholerae* O139 persist in Bangladesh and can cause clinical disease and that a novel disease-causing non-toxigenic O139 isolate also occurs.

## Author Summary

*Vibrio cholerae* serogroup O1 is thought to be the sole causative agent for cholera in Bangladesh and most of the high risk developing countries. Whilst historically *Vibrio cholerae* serogroup O139 has been seen to cause sporadic disease, the overall numbers of reported O139 clinical cases are low, with none reported in Bangladesh since 2005. Here we report four patients suffering from cholera attributed to serogroup O139 *V*. *cholerae*. Cases 1 and 2 were symptomatic (isolated strains 1, 2), and cases 3 and 4 were asymptomatic (isolated strains 3, 4). All cases were from urban Dhaka and represented a range of age groups. Cases 2–4 presented with no sign of dehydration whereas case 1 showed some signs of dehydration. Phenotypic and whole genome sequence data indicates that one of the four O139 *V*. *cholerae* isolates represents a novel O139 subtype. Since natural infection with *V*. *cholerae* O1 or vaccination with currently available licensed cholera vaccines (e.g., Dukoral) provides little protection against O139, we conclude that *V*. *cholerae* O139 remains in circulation and is still causing a low incidence of cholera. Therefore, further studies looking at the significance of these isolates towards the total burden of cholera in Bangladesh is warranted, including clinical evaluation, genome sequencing and immunobiochemistry.

## Introduction

During 1992–93, *V*. *cholerae* O139 was first recognized in Bangladesh, India and other countries in Southeast Asia as a causative agent of epidemic cholera [1–3]. Prior to this, the O1 serogroup was considered the sole cause of cholera epidemics [4]. The isolation of O139 from clinical cases declined quickly after the initial outbreak, with the exception of one epidemic in August 2002 in Dhaka city [5]. Following on from this, isolates of the O139 serogroup were also isolated sporadically from clinical and environmental samples from various regions of Bangladesh during 2005, although no large-scale outbreaks of cholera attributed to O139 serogroup *V*. *cholerae* were reported during this time [5, 6]. Since then, clinical cholera in Bangladesh has been caused entirely by the *V*. *cholerae* O1 serogroup, with an unexplained disappearance of *V*. *cholerae* O139 [5].

With the recent publication of whole genome-based phylogenies of *V*. *cholerae*, we are able to see how the isolates responsible for global cholera relate to each other [7]. It is clear from these data that the isolates causing the current (seventh) pandemic of cholera form a highly related monophyletic lineage dominated by isolates of the El Tor biotype and of the O1 serogroup. This phylogeny, based on whole genome sequence of clinical isolates, shows three overlapping global expansions of *V*. *cholerae* since the pandemic began, denoted wave I, II, and III. From sequencing of a limited number of O139 isolates, it has been shown that they form a single distinct phylogenetic branch that falls within wave II of the seventh pandemic El Tor lineage [7]. At one time, O139 isolates were thought to represent a new lineage of *V*. *cholerae* that would spread globally and perhaps even replace the O1 serogroup. Since the O139 serogroup of *V*. *cholerae* was first recognized, it has been included in cholera surveillance initiatives and in vaccine design efforts [1, 8]. However, pandemic wave II O139 and O1 serogroup isolates are increasingly rare, being replaced almost exclusively by O1 serogroup wave III strains, causing disease globally. Here we report the isolation, characterization and sequence analysis of recent isolates of *V*. *cholerae* O139 recovered from stools of patients hospitalized at the icddr,b diarrheal hospital, as well as from asymptomatic members of the patients’ households. The aim of this study was to compare these new *V*. *cholerae* O139 isolates to existing O139 and O1 isolate sequence data to determine if these new cases were caused by new O139 variants, or the persistence of strains that belong to the known O139 lineage. This will inform the management of future cholera epidemics.

## Materials and Methods

### Study area

This study was carried out in patients presenting to the icddr,b diarrheal hospitals in the Mohakhali and Mirpur neighborhoods of Dhaka, as well as from asymptomatic household members of the cholera patients between December 2013 to March 2014. These patients were enrolled either from the systematic surveillance system for enteric pathogens at the icddr,b Mohakhali hospital, through other ongoing cholera studies [9–11], or through passive surveillance for cholera being conducted at these health facilities as part of a vaccination campaign with oral killed cholera vaccine Shanchol in Dhaka, Bangladesh [11].

### Patient and clinical data

Demographic, socioeconomic, and clinical data were obtained from all study participants. Trained study staff or hospital physicians performed a clinical examination of all study participants. Study participants were assessed for degree of dehydration according to WHO guidelines [12] and treatment was provided according to the icddr,b protocols [13].

### Collection of specimens and characterization of *V*. *cholerae* O139 isolates

All participants gave informed consent for collection of stool/rectal swab specimens. Rectal swabs were collected from case 1 and case 3 because fresh stool specimens were not available. Fresh stool specimens as well as stool swabs were collected from case 2 and case 4. All rectal swabs were placed in a Cary Blair medium and transported to the icddr,b laboratory at room temperature. Two swabs were obtained from each patient. In the laboratory, the first rectal swab taken from each patient was cultured directly on to taurocholate tellurite gelatin agar (TTGA) and the second swab was enriched in alkaline peptone water and incubated at 37°C overnight [14]. After incubation and further culture on TTGA plates, suspected colonies resembling *V*. *cholerae* were tested by slide agglutination with monoclonal antibodies specific for *V*. *cholerae* O1 and O139 [15], as well as by biochemical assays. Specimens that were positive for *V*. *cholerae* O139 were stored at −70°C and later examined by a multiplex PCR assay for concurrent detection of rfb sequences specific for O139/O1 genes of *V*. *cholerae* and for ctxA-specific sequences [16]. Toxigenic *V*. *cholerae* O139 (CIRS 134B) and *V*. *cholerae* O1 El Tor Inaba (strain N16961) and classical Inaba (strain 569B) serotypes were used as positive controls for the multiplex PCR assay. Strains were tested for antimicrobial resistance by disk diffusion method using azithromycin, ciprofloxacin, ceftriaxone, erythromycin, mecillinam, norfloxacine, nalidixic acid, trimethoprim sulfamethoxazole, and tetracycline.

The four *V*. *cholerae* O139 isolates were tested in the rabbit ileal loop assay [17] to detect fluid accumulation and enterotoxicity. *V*. *cholerae* O1 strains 569B and N16961 were used as positive controls. Detection of cholera toxin (CT) was performed by ganglioside GM1-specific enzyme linked immunosorbent assays (ELISA) [18] and differentiation of classical and El Tor biotype CT was made using MAMA PCR described previously [19]. Multiplex PCR assays were performed on a Thermo cycler C-1000 instrument (Bio-Rad). Two sets of oligonucleotide primer pairs were used. The first was O139 rfb-F (5´- AGCCTCTTTATTACGGGTGG-3´), O139 rfb-R (5´-GTCAAACCCGATCGTAAAGG-3´), and the second one was ctxA-F (5´-CTCAGACGGGATTTGTTAGGC-3´), ctxA-R (5´TCTATCTCTGTAGCCCCTATTA-3´); these pairs were used to amplify O139 rfb (amplicon size 449 bp) and ctxA (amplicon size 302 bp) genes respectively using previously described procedures [16]. The product was analysed on 1% agarose gel using Gel Red (BioTium, USA) stain for visualization. Genomic DNA was extracted from eight *V*. *cholerae* strains collected in Bangladesh; Strain 5, Strain 6, Strain 7 and Strain 8 in 1993 and Strain 9 to 12 in 2002 as well as the four *V*. *cholerae* O139 isolates collected in 2013 and 2014. Genomic DNA was prepared by incubating a fresh *V*. *cholerae* colony from a gelatin agar plate into 5 mL of LB broth with overnight shaking at 37°C at 150 rpm. Genomic DNA was extracted with a DNA pure extraction kit (QIAGEN, Germany) according to the manufacturer’s recommendations. Specimen DNA was stored at -70°C and shipped in dry ice to the Wellcome Trust Sanger Institute for sequencing and whole genome analysis.

### Whole genome sequencing and analysis

Isolates were sequenced as multiplexed libraries on an Illumina MiSeq machine, producing 150 nucleotide paired-end reads. Whole genome sequence analysis on the four *V*. *cholerae* O139 isolates collected in 2013 and 2014 was carried out and compared with data from the eight strains collected in Bangladesh in 1993 and 2002 (S1 Table).

The data generated were combined with previously published *V*. *cholerae* genome sequence data [7] from representative O1 seventh pandemic El Tor global wave I, II and III strains, as well as three wave II O139 seventh pandemic El Tor isolates, and used to construct a whole genome single nucleotide polymorphisms (SNP)-based phylogeny. To achieve this, reads for all isolates were mapped to the *V*. *cholerae* O1 El Tor strain N16961 (accession AE003852/AE003853) reference sequence using SMALT v0.7.4 [20], and with GATK for indel realignment [21]. SNPs were called using a combination of SAMtools [22] mpileup and BCFtools as described previously [23]. SNPs falling in regions identified as being recombinant and so not likely to reflect the underlying phylogeny of the bacterium were excluded from this analysis as described in Croucher et al. [24] and a phylogenetic tree was drawn using the non-recombinant SNPS with RAxML [25]. Draft de novo genome assemblies were created using Velvet [26] and scaffolded using SSPACE [27] and Gap Filler [28].

### Accession numbers

1. AE003852/AE003853
2. AAKF03000000
3. ERS452533 –ERS452544
4. ERS013124
5. ERS013125
6. AB012956.1
7. Genes VC_0240 –VC_0264

### Ethical considerations

The hospital surveillance activities of icddr,b were approved by the Research Review Committee (RRC) and Ethical Review Committee (ERC) of icddr,b. According to the icddr,b hospital surveillance system, we only require verbal consent from patients undergoing routine investigation for collecting stool specimens. Consent was documented in the surveillance questionnaire in the hospital surveillance system. Consent was also obtained in accordance with other ongoing studies approved by the RRC/ERC of icddr,b (# PR-10061, # PR-11041)

Based on the above, verbal consent was obtained from one study participant (case 3) while written informed consent was obtained from two cholera patients (cases 1, 2) and one asymptomatic household contact (case 4).

## Results

### Demographic information

The socioeconomic status of the four cases was lower middle class and all were individuals who lived in high risk settings in urban slums in and around Dhaka city. The annual income of the adults (and the parent of the child; case 2) ranged from 15,000–30,000 Taka (~USD 200–300). Two of the participants worked in garment factories while the other two families were self-employed in small businesses. All of the cases reported that they consumed stored tap water in their homes or workplaces and shared kitchens and toilets in the community.

### Symptomatic cases with *V*. *cholerae* O139 infection

#### Case 1

A 26-year old male presented to the icddr,b, diarrheal hospital in Mirpur Treatment center Dhaka, Bangladesh on November 24, 2013 with 12 hours of sudden onset of voluminous diarrhea and vomiting. The patient was a participant in a large cholera vaccine feasibility study. Surveillance was carried out as part of the ongoing hospital-based passive surveillance for cholera. The patient had experienced nine episodes of diarrhea, vomited twice, and ingested a liter of oral rehydration fluid prior to admission. He lived in an urban slum located in Mirpur, in Dhaka. The patient's past medical history was unremarkable. On admission, no other family member of the patient was suffering from severe diarrhea. On examination, the patient was less active, was thirsty, had sunken eyes, dry buccal mucosa, normal skin turgor, normal breathing, and a low volume pulse and showed signs of some dehydration. Other systemic findings were normal. Upon admission, the patient was treated with a single 1 gm oral dose of azithromycin as per icddr,b treatment guidelines. Stool culture, followed by slide agglutination with monoclonal antibodies specific for O139 or O1 indicated *V*. *cholerae* serogroup O139 was the causative agent.

#### Case 2

A 16 month old child was brought to the icddr,b hospital, Mohakhali, Dhaka on March 4, 2014 with 3 days of diarrhea. He had travelled from Savar, 20 km from Dhaka city, to the icddr,b, Mohakhali Hospital for treatment and was enrolled as part of the systematic surveillance system of the icddr,b hospital. Since onset, the patient had experienced several episodes of diarrhea and ingested oral rehydration solution at home. On examination, the patient demonstrated no sign of dehydration and other systemic findings were normal. However, clinical examination showed that the child was undernourished (Z score -1.43). Stool culture tested positive for *V*. *cholerae* and phenotypic tests confirmed the serogroup as O139. No other *V*. *cholerae* serogroup or enteric pathogens were detected.

### Isolation of *V*. *cholerae* serogroup O139 from asymptomatic household contacts of patients with clinically confirmed cholera

#### Case 3

On December 26, 2013, a 52 year old man, a healthy household contact of a cholera positive index case from the cholera vaccine study in Mirpur, Dhaka was diagnosed with *V*. *cholerae* O139 infection. This was detected during a home visit to a cholera positive index case. The patient’s spouse had been admitted to the diarrheal hospital of icddr,b in Mirpur, a week prior to the home visit with cholera caused by *V*. *cholerae* O1. During the home visit, stool specimens were obtained from three household contacts of the index case. Of the three subjects sampled, one was culture positive for *V*. *cholerae* O139. He was asymptomatic with no sign of dehydration and was otherwise clinically healthy.

#### Case 4

A 21 year old female, a household contact of a *V*. *cholerae* O1 infected cholera case was also diagnosed with asymptomatic *V*. *cholerae* O139 infection in 25th February 2014. Her husband had previously been admitted to the icddr,b hospital with a history of 3 days of diarrhea and diagnosed with cholera due to *V*. *cholerae* O1, presenting with severe dehydration. The female household contact was asymptomatic with no other clinical signs of having a diarrheal disease.

### Further characterization of the *V*. *cholerae* O139 isolates: Phenotypic and molecular characteristics

The four *V*. *cholerae* O139 isolates (strains 1–4) collected in this study, having tested strongly positive for the O139 lipopolysaccharide (LPS) O-antigen by the rapid dipstick assay, according to the manufacturer’s recommendations (Span Diagnostics Ltd., India), were further characterized. The serogroup was further confirmed: all four O139 isolates were found to be positive for the O139 *rfb* gene. PCR was used to assay for the presence of the cholera toxin gene, *ctxA*. Strains 2, 3 and 4 were found to be positive for ctxA, all of which when sequenced were characteristic of the classical biotype (see methods). The production and type of toxin was further confirmed by ELISA. The serogroup O139 isolates taken from case 1 (strain 1) was negative for both the classical and El Tor biotype of cholera toxin by both PCR and ELISA. The rabbit ileal loop assay to detect fluid accumulation and enterotoxicity showed that three O139 strains were strongly positive for toxin production (strains 2–4), while the isolate from case 1 (strain 1) failed to induce fluid accumulation and enterotoxicity in the rabbit ileal loop. All four *V*. *cholerae* O139 isolates were found to be resistant to nalidixic acid but were sensitive to all of the other antibiotics tested.

### Whole genome sequence and phylogenetic analysis of the O139 isolates

**Fig 1:**
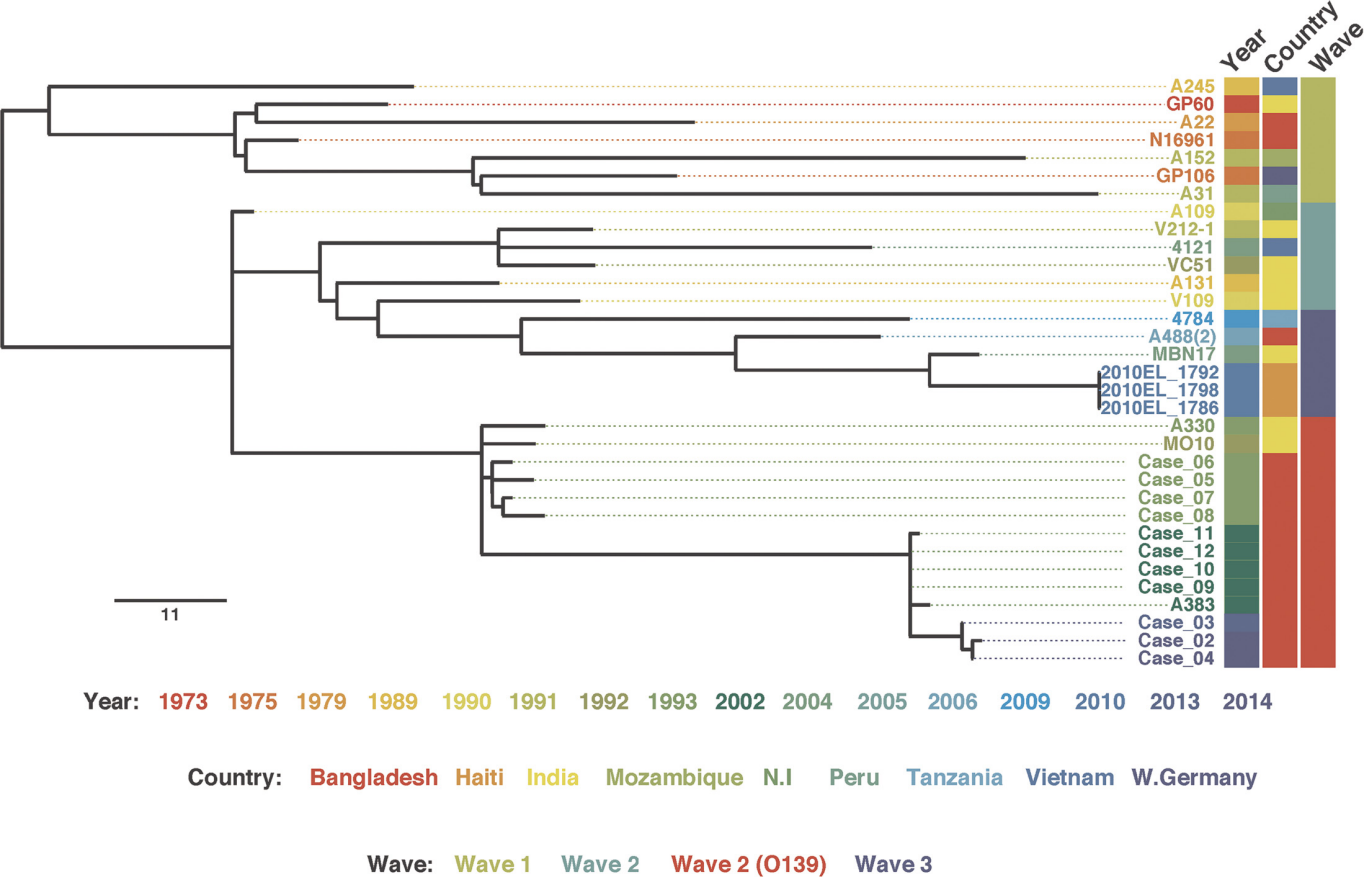
Mid-point rooted maximum likelihood phylogeny of *V*. *cholerae* O139 isolates from this study, excluding the isolate from case 1 (strain 1) but including previously published *V*. *cholerae* isolates. The scale bar represents the number of single nucleotide polymorphisms.

To understand the detailed genetic relationships between the O139 isolates taken from these four patients, we extracted the DNA and sequenced their genomes. For comparison, we also sequenced an additional eight O139 isolates taken in Bangladesh in previous outbreaks in 1993 and 2002. For the isolates obtained from cases 2, 3, and 4, 95.6% of their sequence read data mapped to the genome of the *V*. *cholerae* pandemic 7 strain N16961 serogroup O1 reference sequence (S1 Table). These three genomes differed by between 112–114 (313–316 before removing putative recombination) single nucleotide polymorphisms (SNPs) from the reference sequence. When compared to the O139 strain MO10 (accession AAKF03000000) mapped to the N16961 reference sequence, there were between 46–47 SNPs differentiating MO10 from these three isolates. To determine the phylogenetic relationship of the *V*. *cholerae* O139 isolates we constructed a whole genome core phylogeny from these three *V*. *cholerae* O139 isolates taken from cases 2–4 along with the O139 isolates collected in previous years, and including those previously described [7]. The sequence data are deposited in the European Nucleotide Archive with accessions ERS452533 –ERS452544. The phylogenetic relationships of the isolates sequenced in this study, with the exception of that from case 1, are consistent with previous data [7]. Fig 1 shows that the majority of the isolates fell in the O139 branch of the seventh pandemic El Tor phylogenetic tree, along with the previously published O139 sequences from India and Bangladesh (accessions ERS013124, ERS013125, AAKF03000000) and the majority of isolates sequenced in this study. The new O139 sequences, with the exception of the 2013 isolate from case 1, cluster with a strong temporal signature with isolates in the previous study [7] including the MO10 isolate from India in 1992.

**Fig 2:**
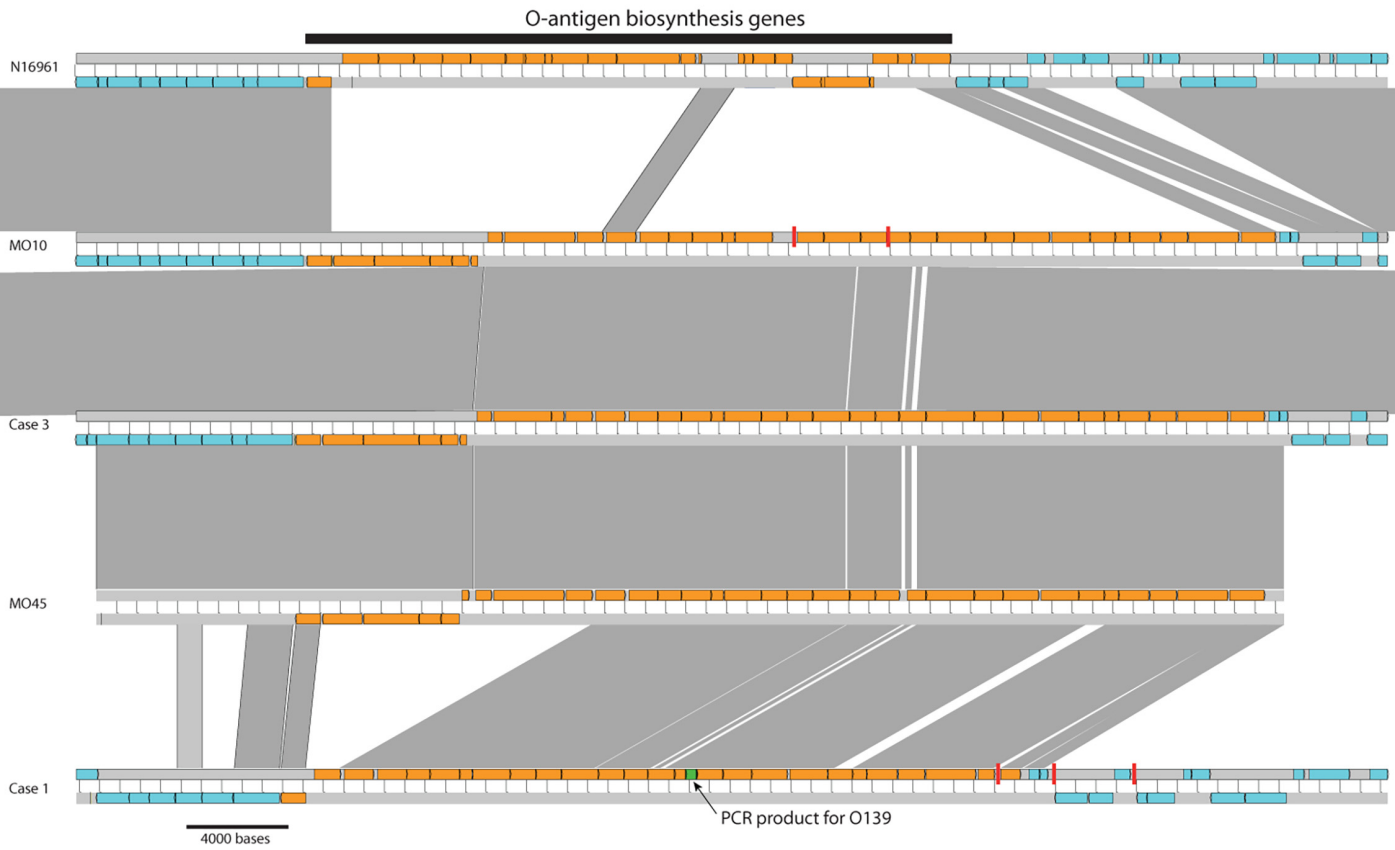
Multi-genome comparison using the Artemis Comparison Tool [29] showing the similarity and synteny of the O-antigen biosynthesis genes (coloured orange) of the O1 N16961 reference sequence (genes VC_0240 –VC_0264), and the draft de novo assemblies of the O139 MO10, the O139 from strain 3 in this study, the O-antigen biosynthesis genes from O139 MO45, and the O139 from strain 1 in this study. Grey blocks indicate genetic similarity; vertical red lines indicate contig breaks in the draft assemblies. The PCR product of the primer sequences used in this study to identify O139 is shown in green in the genome sequence from strain 1.

Whole genome analysis of the non-toxigenic 2013 strain (isolated from case 1) was distinct, with only 83.6% of the sequence reads mapping to the N16961 reference genome, and showing 98,743 and 97,313 (approximately 124,200 and 122,400 before removing putative recombination) SNP differences when compared to N16961 or MO10 using the mapping to the N16961 genome, respectively. The genome sequence data showed that the case 1 isolate (strain 1) lacked the ctxAB genes but possessed the O139 specific rfb gene, confirming the PCR and phenotypic results described above. Based on the mapped genome data, this isolate was highly divergent from all other sequenced O1 and O139 isolates in this study and described previously. Although this isolate was confirmed as belonging to the O139 serogroup using traditional techniques, sequence analysis showed that the genes for the O-antigen biosynthesis genes of this isolate were different from both the O1 and other O139 isolates. To investigate this in greater detail, the 84 contigs of the previously sequenced O139 cholera isolate MO10 were ordered against the O1 N16961 reference using ABACAS [8]. The Artemis Comparison Tool [29] was used to compare the ordered MO10 genome against the case 1 genome; the contiguated sequence of one area of the LPS operon in the case 1 isolate was used to correct the ordering of one of the MO10 contigs. A search in the NCBI database using blastn [30] of the LPS operon sequence of the MO10 isolate found a hit against a 46.7kb sequence of *Vibrio cholerae* genes for O-antigen synthesis, O139 strain MO45 (accession AB012956.1). Comparative sequence analysis of the O139 O-antigen biosynthesis genes in the case 1 isolate showed that it was distinct from those within the O1 N16961 and O139 MO10 isolates, MO45 O-antigen biosynthesis genes, and the O-antigen biosynthesis genes from the genome obtained from case 3 in this study (Fig 2).

## Discussion

We report the isolation and characterization of four isolates belonging to the *V*. *cholerae* serogroup O139 in an eleven month period between December 2013 and March 2014 in Dhaka, Bangladesh. This represents the first report in Bangladesh since 2005 of clinical cases of cholera caused by *V*. *cholerae* O139 infection. This included two patients who presented with acute diarrhea who were ultimately hospitalized.

To put these four isolates in context, icddr,b conducts epidemiological and ecological surveillance for cholera in different parts of Bangladesh. Between 2010–2012, 500 clinical and environmental *V*. *cholerae* strains were isolated, 496 were confirmed as O1 and four as *V*. *cholerae* O139; all of those four previous O139 isolates were obtained from environmental samples [5]. Given the association between *V*. *cholerae* O139 and previous epidemics, the persistence and newly identified sporadic cases of both toxigenic and non-toxigenic *V*. *cholerae* O139 in the environment and in symptomatic and asymptomatic infections is notable, and may have future implications for the diagnosis and prevention of cholera in this region. Recently, a strain of *V*. *cholerae* O139 was isolated from a cholera patient in Beijing in China in May 2014 [31] highlighting the continued low level presence of this lineage in different locales.

Interestingly, in two of our patients, *V*. *cholerae* O139 strains were isolated from asymptomatic household members of *V*. *cholerae* O1 infected cholera patients. In our previous studies, we have shown that household contacts of an index case of cholera are approximately three times more at risk of infection with *V*. *cholerae* [11]. However, we have previously identified non-O1/non-O139 isolates in household members of patients with O1 cholera [32] or even a different O1 Inaba or Ogawa serotype from that isolated from an index case. The *V*. *cholerae* O139 strains presented here were only resistant to nalidixic acid and therefore differ from the *V*. *cholerae* O1 that currently predominate global infections, which are also resistant to trimethoprim-sulfamethoxazole and tetracycline [31].

Whole genome sequence analysis showed that isolates from cases 2–4 fell on the O139 branch of the seventh pandemic El Tor phylogenetic tree, along with the previously published O139 sequences from India and Bangladesh (accessions ERS013124, ERS013125, AAKF03000000) [7]. These data also highlighted the existence of one isolate, from case 1, that was typed genotypically and phenotypically as serogroup O139, but phylogenetically represented a distant non-El Tor pandemic 7 *V*. *cholerae* lineage. By comparing the LPS O-antigen operons it was apparent that this isolate, although highly divergent from the previously sequenced O139 isolates, possessed part of the O139 O-antigen gene cluster both targeted by the diagnostic O139 PCR test and which phenotypically appears sufficient to produce a O139 positive result by ELISA and the rapid dipstick typing methods. Further work will be required to determine fully the significance of this subtype to human health. At the time of writing this report, we had isolated two more strains of *V*. *cholerae* O139 from patients hospitalized with cholera between October and November 2014. These strains are being further characterized at present, and preliminary data suggest that they are phenotypically and genotypically similar to the isolate from case 1.We are at present carrying out detailed analysis of these strains using genomic techniques.

In summary, our data suggest that *V*. *cholerae* O139 strains persist not only within the environment, but also are associated with occasional causes of acute watery diarrhea. Since previous infection with *V*. *cholerae* O1 does not provide protection against O139, and vice versa, our data suggest that O139 could re-emerge as a significant cause of cholera in areas where the pathogen persists. Of note, oral killed cholera vaccine Shanchol is bivalent, and contains components of both O1 and O139 organisms, while other currently commercially available cholera vaccines are monovalent, providing protection against O1 alone. We are continuing with the surveillance of patients with acute watery diarrhea for detection of *V*. *cholerae* O139 to monitor emergence of new variants and also to detect any new and reemerging outbreaks or epidemics using microbiological and genomic analysis. This is extremely important for planning future strategies for immunoprophylactic preventive measures.

## Supporting Information

S1 Table Genome sequencing data of O139 isolates (1993–2014). (DOCX)
