# Supplementary material for: Vibrio cholerae Serogroup O139: Isolation from Cholera Patients and Asymptomatic Household Family Members in Bangladesh between 2013 and 2014
Source: PLoS Negl Trop Dis. 2015 Nov 12;9(11):e0004183. doi: 10.1371/journal.pntd.0004183 (PMC4642977; doi:10.1371/journal.pntd.0004183)
Supplement: S1 Table — (DOCX) [file pntd.0004183.s001.docx]

**Supplementary Table 1**

| Sample | ERS number | ERR number | Number of reads | Total bases | Read length | Mean insert size | Mapped reads to N16961 | N16961 reference length | Mean coverage depth | Percent reads mapped to N16961 reference | Assembly size | Number of contigs |
| --- | --- | --- | --- | --- | --- | --- | --- | --- | --- | --- | --- | --- |
| Case_01 | ERS452533 | ERR568405 | 1895148 | 284272200 | 150 | 473 | 1594926 | 4033501 | 59.3 | 83.6 | 4014127 | 117 |
| Case_02 | ERS452534 | ERR568406 | 1794616 | 269192400 | 150 | 492 | 1702590 | 4033501 | 63.3 | 95.6 | 4011747 | 44 |
| Case_03 | ERS452535 | ERR568407 | 1907124 | 286068600 | 150 | 477 | 1808006 | 4033501 | 67.2 | 95.6 | 4015546 | 36 |
| Case_04 | ERS452536 | ERR568408 | 1788604 | 268290600 | 150 | 476 | 1693735 | 4033501 | 63.0 | 95.6 | 4011017 | 53 |
| Case_05 | ERS452537 | ERR568409 | 2483580 | 372537000 | 150 | 480 | 2348989 | 4033501 | 87.4 | 95.7 | 4022602 | 35 |
| Case_06 | ERS452538 | ERR568410 | 1928154 | 289223100 | 150 | 508 | 1815269 | 4033501 | 67.5 | 95.7 | 4022975 | 44 |
| Case_07 | ERS452539 | ERR568411 | 1916828 | 287524200 | 150 | 482 | 1809947 | 4033501 | 67.3 | 95.7 | 4028921 | 39 |
| Case_08 | ERS452540 | ERR568412 | 1951022 | 292653300 | 150 | 487 | 1834571 | 4033501 | 68.2 | 95.7 | 4030764 | 39 |
| Case_09 | ERS452541 | ERR568413 | 2117620 | 317643000 | 150 | 487 | 2008904 | 4033501 | 74.7 | 95.7 | 4015391 | 33 |
| Case_10 | ERS452542 | ERR568414 | 2025182 | 303777300 | 150 | 472 | 1921626 | 4033501 | 71.5 | 95.7 | 4014046 | 46 |
| Case_11 | ERS452543 | ERR568415 | 2377624 | 356643600 | 150 | 466 | 2256289 | 4033501 | 83.9 | 95.7 | 4016601 | 33 |
| Case_12 | ERS452544 | ERR568416 | 2125910 | 318886500 | 150 | 493 | 2018965 | 4033501 | 75.1 | 95.7 | 4016908 | 32 |
